# Improving Awareness of Mental Health Services Among International Medical Graduates (IMGs) in Psychiatry Training in the West Midlands: A Quality Improvement (QI) Project

**DOI:** 10.1192/bjo.2026.11548

**Published:** 2026-06-30

**Authors:** Omotola Ogunjobi, Ajay Baskaran, Asma Javed

**Affiliations:** Black Country Healthcare NHS Foundation Trust, Dudley, United Kingdom

## Abstract

**Aims:**

The NHS mental health system is complex, with a wide range of community, inpatient, specialist, and population-specific services. New trainees in psychiatry, especially IMGs, often begin work with variable knowledge of available services and limited understanding of who does what across pathways. This can impact confidence, appropriate signposting, multidisciplinary working, and patient care.

The aim of this QI project was to assess baseline knowledge of NHS mental health services among IMGs and to develop a clear, accessible resource to improve understanding of service roles and pathways.

**Methods:**

An initial survey using Microsoft Forms was distributed to IMG psychiatry trainees across West Midlands. Participants were asked how long they had worked, to rate their knowledge of NHS mental health services when they first started (on a 1–5 scale: no clue, rough idea, neutral, pretty good, excellent), and to list any services they were aware of at that time.

Quantitative data were analysed descriptively, and free-text responses were thematicallyreviewed to identify patterns in awareness and gaps in understanding. Based on the findings, a practical resource titled “Mental Health Services; Who Does What?” was developed.

**Results:**

Respondents reported working in NHS mental health roles for between 2 and 68 months, with a mean duration of 23 months. When asked to retrospectively rate their knowledge of NHS mental health services, out of 11 respondents, 3 selected “no clue”, 5 selected “rough idea”, 1 “neutral”, 1 “pretty good”, and 1 “excellent.”

Free-text responses revealed a wide variation in awareness. Most participants were familiar with core services such as Community Mental Health Teams, inpatient services, crisis and home treatment teams, and liaison psychiatry. Awareness of more specialist services such as assertive outreach, neuropsychiatry, perinatal, eating disorder services, forensic services, and voluntary sector support) was inconsistent and often superficial, with limited understanding of specific roles and responsibilities.

**Conclusion:**

This project highlights significant variation and overall low confidence in IMG trainees' knowledge of NHS mental health services at the start of employment. The development of a concise, structured service guide directly addresses identified gaps and provides a practical tool to support induction and early learning. In the future, we will collate feedback and measure improvements in confidence and service awareness following implementation of the guide.

